# Recent Advances and Future Perspectives on Microfluidic Liquid Handling

**DOI:** 10.3390/mi8060186

**Published:** 2017-06-12

**Authors:** Nam-Trung Nguyen, Majid Hejazian, Chin Hong Ooi, Navid Kashaninejad

**Affiliations:** Queensland Micro- and Nanotechnology Centre, Nathan Campus, Griffith University, 170 Kessels Road, Brisbane, QLD 4111, Australia; majid.hejazian@griffithuni.edu.au (M.H.); chinhong.ooi@griffithuni.edu.au (C.H.O.); n.kashaninejad@griffith.edu.au (N.K.)

**Keywords:** continuous microfluidics, micromixers, cell separation, digital microfluidics, liquid marbles, electrowetting-on-dielectric (EWOD), microfluidic liquid handling

## Abstract

The interdisciplinary research field of microfluidics has the potential to revolutionize current technologies that require the handling of a small amount of fluid, a fast response, low costs and automation. Microfluidic platforms that handle small amounts of liquid have been categorised as continuous-flow microfluidics and digital microfluidics. The first part of this paper discusses the recent advances of the two main and opposing applications of liquid handling in continuous-flow microfluidics: mixing and separation. Mixing and separation are essential steps in most lab-on-a-chip platforms, as sample preparation and detection are required for a variety of biological and chemical assays. The second part discusses the various digital microfluidic strategies, based on droplets and liquid marbles, for the manipulation of discrete microdroplets. More advanced digital microfluidic devices combining electrowetting with other techniques are also introduced. The applications of the emerging field of liquid-marble-based digital microfluidics are also highlighted. Finally, future perspectives on microfluidic liquid handling are discussed.

## 1. Introduction

In recent years, the technology of microfluidics has progressed rapidly and become an integral part in many engineering and biomedical applications [1]. Microfluidics has been regarded as the main driver for the paradigm shift in four main areas: molecular analysis, biodefence, molecular biology and microelectronics [2]. The integration of microfluidic components into a single chip led to the advent of lab-on-a-chip (LOC) [3], micro total analysis system (µTAS) [4] and point-of-care (POC) diagnostic devices [5]. In such devices, as well as most biological processes, liquid handling is of great importance, as its quality can significantly affect the end results. According to the way a small liquid amount is handled and manipulated, the field of microfluidics is further classified as continuous-flow microfluidics and digital (droplet-based) microfluidics.

Continuous-flow microfluidics requires an external means to deliver the continuous flow of a single liquid phase or multiple phases through microchannels [6]. The two major and opposing fluid handling tasks of continuous-flow microfluidics are mixing and separation. In particular, mixing of reactants is required to initiate the interactions involved in biological processes such as protein folding and enzyme reactions [7]. For instance, in tumor-on-a-chip microfluidic platforms [8], mixing and delivery of a combination of drugs are necessary. Separation also plays an important role in sample preparation for both analytical chemistry and biological applications [9]. Additionally, cell sorting and separation need to be carried out precisely to develop microfluidic disease models and POC diagnostic tools. Yet, using continuous flow microfluidic technology for mixing and separation seems paradoxical. On the one hand, the high surface-to-volume ratio in microfluidics reduces the required sample, and is ideal for biological, biochemical and pharmaceutical applications. On the other hand, the dominant laminar and low-Reynolds-number flow regime delays the mixing and separation process, and requires a larger mixing and separation length scale. This problem indicates the need for innovative mixing/separation methods, especially for LOC applications, where a number of components need to be integrated on a single chip. Traditionally, these methods are categorised as passive (without external energy) and active (in the presence of external energy) techniques [7].

The advantages and disadvantages of passive methods, which utilise chaotic advection to reduce the mixing time, were extensively reviewed by Suh and Kang [10]. The operation principles and mixing capabilities of a broad range of predominantly used micromixers were reviewed by Lee et al. [11]. Ward and Fan [12] categorised and discussed a variety of basic passive microfluidic mixing enhancement techniques, such as slanted wells/pillars, multiphase mixing enhancement and active enhancement techniques, such as thermal enhancement, acoustic waves and flow pulsation. A number of review articles addressed the current state of microfluidic separation techniques. For instance, Sajeesh and Sen presented a comprehensive review on different microfluidic passive and active techniques for particle separation and sorting [9]. In cell biology, microfluidic methods that do not require biochemical labels to isolate and identify cells are referred to as label-free techniques, and have attracted a great deal of attention. Gosset et al. reviewed label-free microfluidic techniques that use the intrinsic properties of the cell, such as its size and other physical signatures [13]. Microfluidic techniques can also be used for detection and separation of cancer cells. Chen et al. [14] discussed high-throughput microfluidic techniques, such as cell-affinity micro-chromatography and magnetically activated sorting. Shields et al. presented recent advances in microfluidic cell separation, along with the challenges in the commercialisation of such devices for practical clinical applications [15].

Combining microfluidics with the science of emulsion, digital microfluidics (DFM) has been developed as a technology dealing with the manipulation of individual droplets, rather than continuous streams of liquid [16]. This field has numerous applications and has the potential to revolutionise various biochemical and biomedical protocols, as well as cell-based assays [17]. DMF has numerous advantages, such as minimum reagent requirement, fast response rates, and more importantly, the capability of performing several parallel procedures [18]. These advantages make DMF an ideal candidate for practical LOC and POC diagnostic devices in clinical use [19]. However, there are still many challenges that need to be addressed in this field, such as droplet evaporation, droplet handling techniques, material selection, etc. [20]. A few recent review articles exist in this emerging field. Samiei et al. [21] reviewed the recent advances in DMF regarding fabrication technology, handling of biological reagents, packaging and portability. Using magnetic actuations to handle the individual droplet is also of great interest. Possibilities and challenges of magnetic digital microfluidics were recently reviewed by Zhang and Nguyen [22].

**Figure 1:**
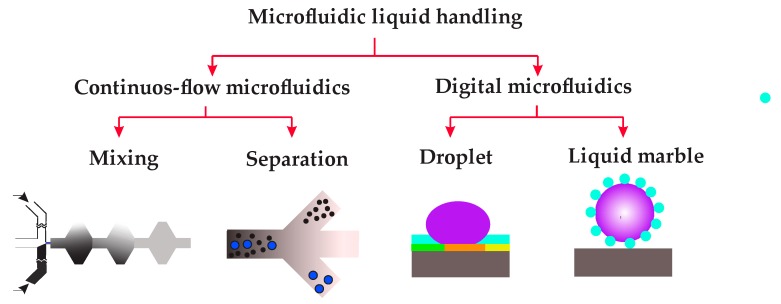
Scope of the present review. Microfluidic liquid handling are important parts of biological processes that can be divided into continuous-flow microfluidics and digital microfluidics. Mixing and separation are two common liquid handling techniques in continuous-flow microfluidics, whereas droplet-based and liquid-marble-based digital microfluidic technologies are used for manipulating discrete droplets.

The scope of the present review paper is summarised in Figure 1. The first part of this paper discusses recent advances of continuous-flow microfluidics in liquid handling, i.e., mixing and separation. In particular, recent progress regarding two key mixing enhancement techniques, namely external forces and complex geometry, are revisited. Subsequently, the paper discusses continuous-flow microfluidic separation techniques such as magnetofluidics, inertial microfluidics, acoustofludics, dielectrophoretics and optofludics. The second part of this paper mainly deals with the advances in both droplet-based DMF and liquid-marble-based DMF. This part discusses the most common methods of droplet-based DMF, such as electrowetting-on-dielectric (EWOD), dielectrophoresis, and magnetic techniques to dispense, move or mix droplets. Finally, the promising field of liquid-marble-based DMF, along with its application as a microbioreactor to culture three-dimensional tissues, will be highlighted.

## 2. Continuous Flow Microfluidics

### 2.1. Mixing

Mixing is an essential step in most lab-on-a-chip platforms, as sample preparation is required for a variety of biological and chemical assays. Diffusion-based mixing techniques fail to satisfy the recent demand for rapid and homogeneous mixing. Various strategies have been implemented to enhance the efficiency of continuous-flow microfluidic mixing. In this section, we present the recent advances in continuous mixing with microfluidics.

#### 2.1.1. Mixing with External Energy Sources

**Figure 2:**
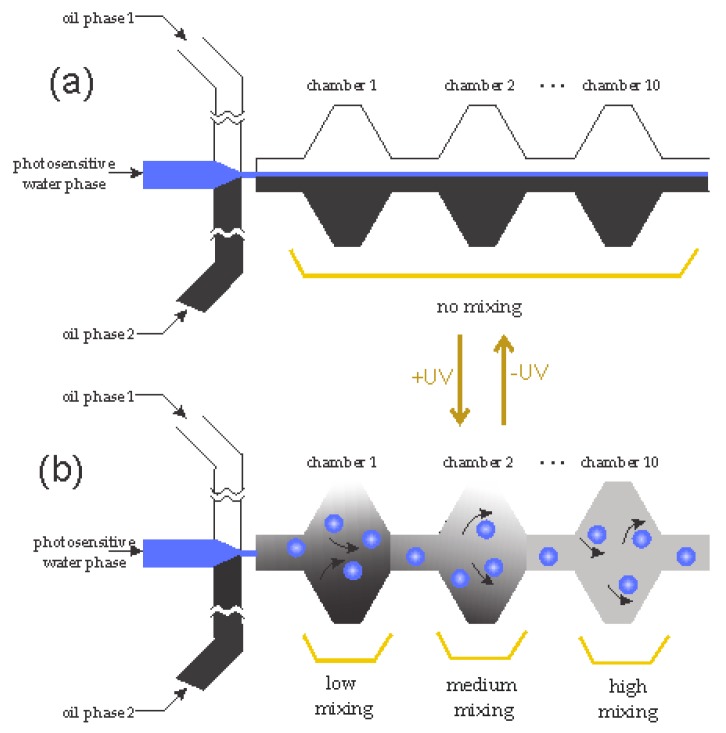
Schematic of the reversible optofluidic mixer developed by Venancio-Marques et al. [28]: (**a**) when the ultraviolet (UV) is off, the two oil phases are not mixed together; (**b**) at the presence of UV, the photosensitive water turns into the droplets, causing the mixing between two oil phases. Adapted from [28].

One of the strategies for increasing mixing efficiency is employing external energy sources to create disturbances, such as acoustic, magnetic, electrostatic. Mass transport of a species in a superparamagnetic solution can be enhanced with an external magnetic field [23]. Utilising embedded electromagnets for magnetofluidic actuation, Mao and Koser [24] demonstrated that the mixing of two streams can be significantly improved. Hejazian and Nguyen [25] proposed a rapid and efficient micromixer using a permanent magnet and a magnetic fluid. The permanent magnet induces a non-uniform magnetic field, and correspondingly, a secondary flow, that mixes a non-magnetic stream with another stream containing diluted ferrofluid. Workamp et al. [26] presented a microfluidic suspension-based mixer with low pressure drop. The mixer consists of a chamber where particles are driven by a moving magnet. Peng et al. [27] proposed a micromixer based on parallel manipulation of individual magnetic microbeads. Rotating magnets generate a circular motion of magnetic beads. As a result, local vortices are created across the microchannel, leading to efficient mixing. Venancio-Marques et al. [28] demonstrated optofluidic mixing in a microfluidic device. As shown schematically in Figure 2, the system consists of three streams, a photosensitive water stream sandwiched between two oil phases. Without light illumination, the flow system is a typical flow-focusing configuration [29]. Light illumination generates water micro-droplets that stir and mix the two continuous oil streams.

**Figure 3:**
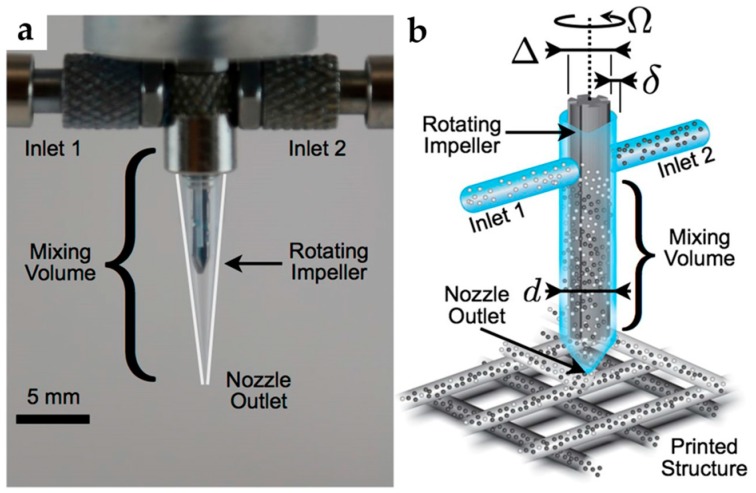
Impeller-based active mixer developed by Ober et al. [30]: (**a**) optical image of the mixer; (**b**) representation of the mixing nozzle. Reproduced with permission (granted by PNAS for non-commercial purposes) from [30].

Ober et al. [30] examined a rational framework for designing microfluidic active mixers, Figure 3. The micromixers were 3D printed and integrated with a rotating impeller. The capability of continuous mixing of complex fluids was demonstrated. Furthermore, the relationships between mixer dimensions and operating conditions were verified experimentally.

Cui et al. [31] proposed a microfluidic mixer based on acoustically induced vortices created by localized ultrahigh frequency (UHF) acoustic fields. A UHF piezoelectric resonator (SMR) was capable of generating powerful acoustic streaming vortices, resulting in efficient mixing. The authors reported homogeneous mixing, with 87% mixing efficiency at a Peclet number of 35,520, within just 1 ms. Fang et al. [32] proposed a micromixer with a streamline herringbone structure, based on total glass. High direct current (DC) voltage-activated migration condition was applied to the microfluidic device as well, and the performance of the mixer was investigated. They reported an efficiency of over 90% in 20 mm, in a mixing channel of only 300 nL. Shang et al. [33] explored a vortex generated by an acoustic actuator within a circular chamber to improve mixing. The strength of the vortex was tuned by the applied voltage. Their research thus showed that mixing efficiency can be increased by adjusting the voltage.

#### 2.1.2. Mixing with Complex Geometries

**Figure 4:**
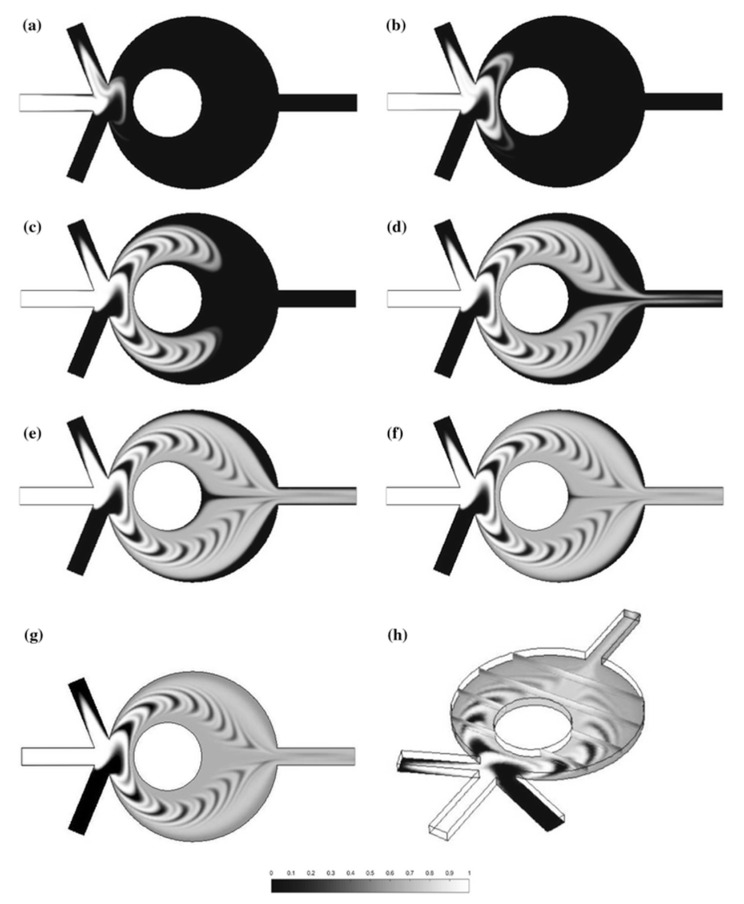
Fast response and geometrically scalable micromixer proposed by Cortelezzi et al. [35]: (**a**–**g**) two-dimensional representation of concentration distribution when time evolves form 2.6, 3.6, 7.6, 11.6, 19.6, 39.6 to 199.6 s, respectively; (**h**) three-dimensional representation of the concentration distribution at 199.6 s. Reproduced with permission from the original in study [35].

Using external actuations to increase the mixing efficiency could be expensive and challenging [29]. Another alternative technique for increasing the mixing efficiency is utilizing relatively complex geometries for chaotic advection. As the flow regime in most microfluidic systems is laminar, the quality of mixing is highly dependent on chaotic advection induced by the geometry of the microchannel. Wu and Nguyen [29] evaluated, both analytically and experimentally, the mixing efficacy of a rectangular microchannel using two-phase hydraulic focusing. To that end, two streams of sheath flow were used to hydraulically focus two streams of sample flow. Their results showed that the focusing ratio was a function of both viscosity ratio and flow rate of sheath and sample flows. To further enhance the mixing efficiency, Nguyen and Huang [34] combined the hydrodynamic focusing technique with time-interleaved segmentation. The results of the paper revealed that, while hydrodynamic focusing could reduce the transversal mixing path, sequential segmentation could also be used to decrease the axial mixing path. It was found that switching frequency and average flow velocity also affected the mixing quality. Cortelezzi et al. [35] proposed a geometrically scalable micromixer capable of achieving fast mixing over a wide range of operating conditions. As shown in Figure 4, the mixer consists of a cylindrical mixing chamber and a cylindrical obstacle. With alternate switching of the inlets to create time-interleaved segmentation, the mixer could reach an efficiency of about 90.8%.

**Figure 5:**
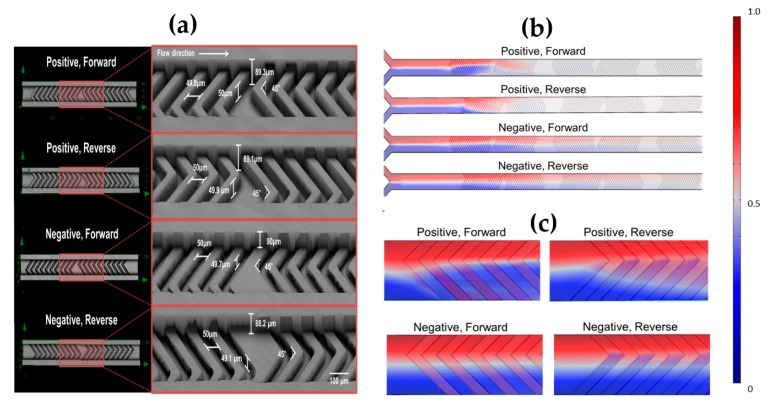
The staggered herringbone mixer (SHM) created by Kwak et al. [36]. (**a**) Detailed pattern structures and flow directions; (**b**) Mixing quality after 2.5 cycles in positive and negative SHM structures subject to both forward and reverse flow directions; (**c**) Top view images of four different SHMs indicating the mixing efficiency at the beginning of the first cycle. Red and blue colors correspond to fluorescence dye and water, respectively, while white color indicates complete mixing. Reproduced with permission (under Creative Commons Attribution (CC BY) license) from [36].

Kwak et al. [36] proposed the use of a positive repeated pattern of a staggered herringbone mixer (SHM) in a microchannel to improve mixing efficiency, and compared the results with those obtained from the negative pattern of SHM. It was found that the mixing efficacy would be higher if positive SHM and/or forward flow were used. In particular, a positive pattern SHM could reach completed mixing after two cycles with both forward and reverse flows, while four and five cycles were needed for complete mixing in the negative pattern SHM with forward and reverse flow directions, respectively, Figure 5.

Salieb-Beugelaar et al. [37] presented microfluidic 3D helix mixers for controlled chemical reactions. The authors created the complex channel geometry with thread embedded in polydimethylsiloxane (PDMS). The threads created double helix and triple helix structures in the same device.

Adam and Hashim [38] reported the design and the fabrication of a micromixer with short turns and showed that it could reach a mixing efficiency of 98% at Reynolds number less than 2. Sivashankar et al. [39] proposed a micromixer with a twisted structure to enhance mixing. The 3D microfluidic mixer was fabricated by laser micromachining. The results showed that good mixing can be achieved with more than three mixing units. Wang et al. [40] used triangle baffles embedded in a microchannel to enhance mixing. The simulation results show that mixing efficiency can be improved by increasing the apical angle of the triangles from 30° to 150°. Lehmann et al. [41] performed continuous recalcification of citrated whole blood using a microfluidic herringbone mixer. A herringbone structure was fabricated on top of the channel to generate transverse flows within the microfluidic channel.

Plevniak et al. [42] demonstrated a 3D printed microfluidic mixer for fast mixing of reagents with blood through capillary force. The device was integrated with a smartphone for the point-of-care diagnosis of anemia from a finger-prick blood sample. The results obtained with the device are in line with clinical measurements. Li et al. [43] proposed a microfluidic mixer consisting of an irregular Y junction followed by an observation channel. The mixer was ultra-rapid, as complete mixing was achieved with a mixing time of just 5.5 μs. The authors interrogated the hairpin formation in the early folding process of human telomere G–quadruplex.

### 2.2. Separation

In the last two decades, significant advances have been made in the development of continuous-flow microfluidic separation. With continuous injection and collection of samples, a high separation throughput can be achieved. Moreover, continuous-flow microfluidic separation also has the benefit of real-time monitoring, and the potential for the integration with other continuous-flow processes [44]. Based on the unique signature of the sample components, a suitable external force can be chosen for the separation process. The separation of particles and cells can employ a variety of external forces such as hydrodynamic, electrophoretic, dielectrophoretic, magnetophoretic, acoustic, and inertial force [45]. In this section, we explore the current range of continuous separation methods.

#### 2.2.1. Magnetofluidic Separation

Continuous-flow magnetofluidic separation has recently gained considerable interest from the research community. Due to the contactless nature of magnetic force, magnetofluidic methods do not alter the pH level or the temperature of the sample, and as a result, it has no negative effect on the viability of cells [45,46,47]. Magnetofluidic separation of cells and particles is categorised into two main concepts: positive and negative magnetophoresis. If the magnetic susceptibility of the medium fluid is higher than that of the particles, negative magnetophoresis occurs, and vice versa. Over the last decade, a number of reviews have been published on magnetofluidics, reporting a diverse range of techniques for separation of particles and cells, based on negative and positive magnetophoresis [47,48,49,50,51]. Superparamagnetic carrier fluids, such as ferrofluid, create a secondary flow towards the source of a magnetic field. This phenomenon is called magnetoconvection [23,52]. Exploiting magnetoconvection, a highly size-sensitive separation of microparticles was achieved within a microchannel [53]. Using two arrays of attracting magnets, non-magnetic polystyrene micro-particles were captured in different locations along a straight microchannel. Applying a similar concept, Zhou et al. [54] introduced a platform for simultaneous capture of non-magnetic and magnetic particles. For this purpose, an external magnetic field was generated with a permanent magnet positioned next to a T–junction in the microchannel.

Particle focusing with magnetofluidics has been reported using two sets of repelling magnets [49,51,55]. Liang and Xuan [56] reported sheathless focusing of non-magnetic particles. A T–microchannel, a single permanent magnet, and diluted ferrofluid as the superparamagnetic carrier fluid, were used for this purpose. A relatively strong magnetic field gradient should be implemented to achieve high efficiency and size sensitivity. For instance, decreasing the distance between the external magnetic field source and the fluidic channel is a solution for increasing the magnetic field gradient. Zhou and Wang [57] introduced a convenient and low-cost technique for the enhancement of magnetic field gradient. For this purpose, a prefabricated channel was formed next to the microfluidic channel. A mixture of iron powder and polydimethylsiloxane (PDMS) was injected into the channel. The iron–PDMS structures were placed just a few microns from the microchannel. Separation of nanoparticles with magnetofluidics has also recently gained attention. Wu et al. [58] proposed an efficient method for size-selective separation of magnetic nanospheres using a magnetofluidic device. Two monodisperse nanosphere samples (90 nm and 160 nm) were successfully separated from the polydispersing particles solution, with varied particle diameters from 40 to 280 nm.

#### 2.2.2. Inertial Microfluidics

**Figure 6:**
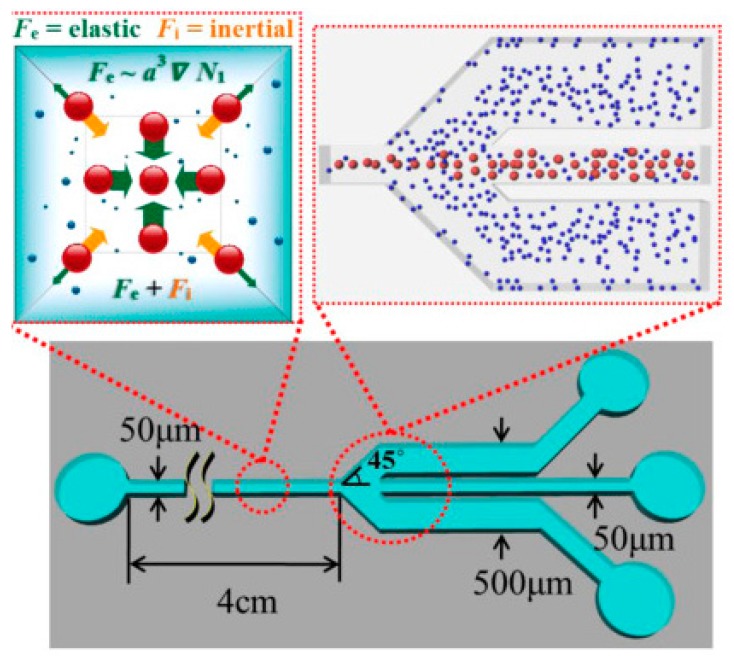
Microfluidic separator based on inertial microfluidics with its working principles developed by Ahn et al. [64]. Reproduced with permission from [64].

Inertial microfluidics is another emerging field of continuous-flow particle separation. Inertial microfluidics is a suitable method for rare cell sorting, due to such various advantages as high throughput, simplicity, precise manipulation and low cost [59,60]. A number of reviews have summarised the existing techniques and designs of inertial microfluidics [59,60,61,62,63]. The inertial force is often combined with other forces, such as hydraulic, magnetic, centrifugal, or hydrodynamic forces, in order to obtain a higher separation efficiency. Ahn et al. [64] designed a sheathless elasto-inertial focusing microfluidic separator, and performed a systematic study evaluating the parameters affecting the performance of a microfluidic separator based on inertial microfluidics. The schematic illustration along with the working principles of their fabricated microfluidic separator is shown in Figure 6.

Optimisation parameters, such as particle concentration and flow rate, as well as the effect of particle–particle interaction in the separation process, were determined [65]. Combining lift forces and Dean flow drag forces, algae species were separated, based on their shape and size, in a spiral microchannel. Monoraphidium species was successfully separated from the differently shaped Cyanothece, with 77% separation efficiency.

Zhou et al. [66] demonstrated a hybrid method based on the combination of inertial microfluidics and magnetofluidics for size-selective separation of micro-particles. Spherical diamagnetic polystyrene particles of 10 μm and 20 μm were successfully separated using this technique. Clime et al. [67] furthermore demonstrated filtration and extraction of pathogens from food samples, utilising hydrodynamic focusing and inertial lateral migration. The microfluidic platform was capable of removing up to 50% of debris from ground beef samples.

#### 2.2.3. Acoustofluidic Separation

**Figure 7:**
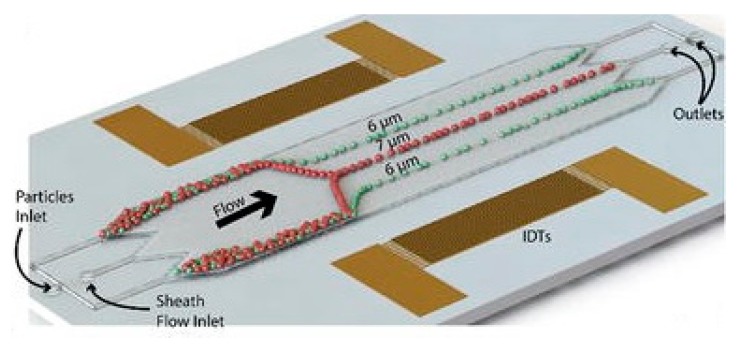
Schematic illustration of flow-rate-insensitive device for continuous particle sorting, designed by Ng et al. [76]. Reproduced with permission from [64,76].

The use of acoustic waves is another technique that has been used for continuous particle separation with microfluidics. Because of such advantages as simplicity of design, low-cost, and biocompatibility due to its contactless nature, acoustic wave devices have been integrated with microfluidic devices. A number of recent reviews reported on the different configurations of acoustofluidic devices [68,69,70,71,72,73]. Mathew et al. [74] developed a two-dimensional dynamic model for tracing the path of microparticles in continuous-flow microfluidics employing acoustic waves. The effect of parameters, such as acoustic energy density and initial vertical location, on the displacement of microparticles were examined with this model. Shields et al. [75] designed a multi-stage microfluidic platform for separation of cancer cells from blood. In the first module, the acoustic standing wave is exploited for immediate alignment of cells. Magnetic separation techniques then purify and capture individual cells for on-chip analyses, in the next two steps. Ng et al. [76] designed a flow-rate-insensitive device for continuous particle sorting, Figure 7. The device uses surface acoustic waves that combine both standing and travelling wave components to create pressure nodes. The particles were trapped in locations with a stable pressure based on their size, and separated through a distinct exit.

#### 2.2.4. Dielectrophoretic Separation

Dielectrophoretic method has been another area of interest for continuous particle separation with microfluidics in recent years. The use of dielectrophoretic force with microfluidics for continuous particle separation has advantages such as low cost, rapidity, size sensitivity, and selectivity. Previously published reviews discuss a variety of techniques used for dielectrophoretics-based cell and particle separation [77,78,79,80,81]. Cui et al. [82] proposed a dielectrophoresis (DEP)-based method for size-based particle separation. The authors demonstrated the extraction of larger particles, retaining small particles, and also eluting mid-size particles using pulsed dielectrophoresis. Kim et al. [83] proposed an integrated Dielectrophoretic–Magnetic Activated Cell Sorter (iDMACS). The target cell types were sorted based on surface markers, via specific receptor–ligand binding to either DEP or magnetic tags. The device could achieve 900-fold enrichment of multiple bacterial target cell types, with over 95% purity after a single round of separation. Yang et al. [84] examined dielectrophoresis (DEP)-active hydrophoresis for sorting particles and cells. The device consists of prefocusing and sorting steps, and achieved highly efficient and pure separation of both viable and nonviable Chinese Hamster Ovary (CHO) cells from medium fluid.

#### 2.2.5. Optofluidic Separation

**Figure 8:**
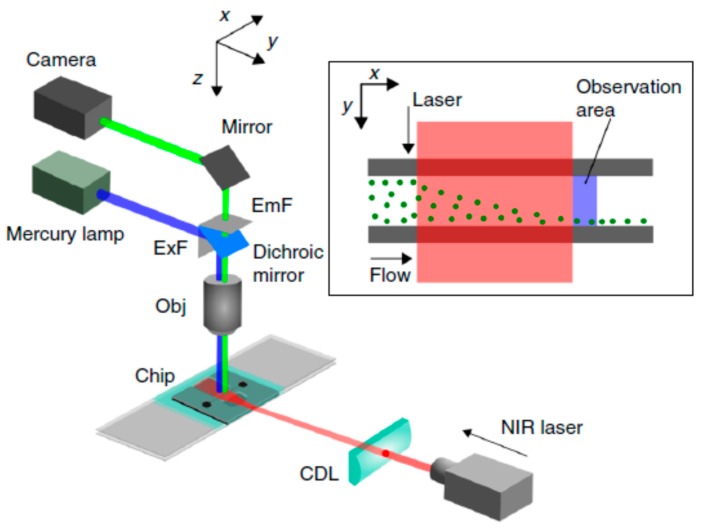
The experimental test rig of microfluidic optical radiation pressure for particle separation developed by Kotari et al. [85]. The SU–8 layer on the microchip guides the near-infrared (NIR) laser beam through the lens. Reproduced with permission from original in study [85].

Kotari et al. [85] exploited optical radiation pressure for particle separation in a microfluidic device. Figure 8 illustrates the experimental setup for lateral particle sorting which uses SU–8 as a waveguide to irradiate a near-infrared (NIR) laser beam to facilitate the observation of particle distribution. Using scattering force, particles are manipulated corresponding to the amount of light received by them. Polystyrene beads were successfully transported by the optical scattering force with an energy density of less than 10 mW/mm^2^.

### 2.3. Advanced Continuous-Flow Microfluidics with Combined Mixing and Separation

For many biological and chemical analyses, mixing of reagents, and subsequent separation from the remaining sample and vice versa, are the main reasons for making these analyses labour-intensive, time-consuming, expensive and cumbersome. The unique feature of microfluidics is that it allows for the integration of both mixing and separating components on a single chip. In addition, incorporating gas-permeable PDMS membranes into such microfluidic platforms allows for the fabrication of advanced microbioreactors, capable of performing a variety of chemical and biological processes. Specialised POC diagnostic platforms, such as lab-on-a-disc, show great promise for fast, reliable and cost-effective immunoassay tools. For example, the lab-on-a-disc platform developed by Kuo and Li [86] allowed for the separation of plasma from whole blood in only six seconds. Subsequently, the plasma-free blood was able to be mixed with related reagents for other diagnostic tests. The microfluidic device for the prothrombin time (PT) test was 15 times faster than the conventional bench-top counterpart. For both diagnostic and therapeutic purposes, high-throughput label-free microfluidic cell sorters are in great demand. Using the passive hydrodynamic approach, Tallapragada et al. [87] proposed a scale-independent method to separate and encapsulate inertial particles, specifically pancreatic islets, in serpentine microchannels. Finally, microfluidic chromatographic platforms have also opened up new avenues for separation chemistry, especially for protein purification [88].

## 3. Digital Microfluidics

Digital microfluidics (DMF) involves the manipulation of small, discrete droplets, usually in the microlitre scale or smaller. The main tasks of DMF involve dispensing droplets, moving droplets, merging droplets or mixing contents within a droplet. Numerous techniques have been developed to perform these tasks, as elaborated on in extensive recent reviews [20,21,22,89,90,91].

### 3.1. Droplet-Based DMF

DMF devices can have a basic open planar form, where the droplet is placed on a solid planar surface. The plate is usually engineered to provide an energy gradient to drive the droplet. In some cases, a top plate is added to facilitate control of the sandwiched droplet. With proper design, droplets can be moved across the plate in two dimensions. However, the droplet can also be further controlled by constructing channels between the plates, thus restricting the droplet to a one-dimensional movement. The immiscible fluid surrounding the droplet maintains the separation of droplets. Specially treated surfaces in contact with the droplet minimises loss of liquid during transport.

#### 3.1.1. Electrowetting-on-Dielectric (EWOD) Technique

**Figure 9:**
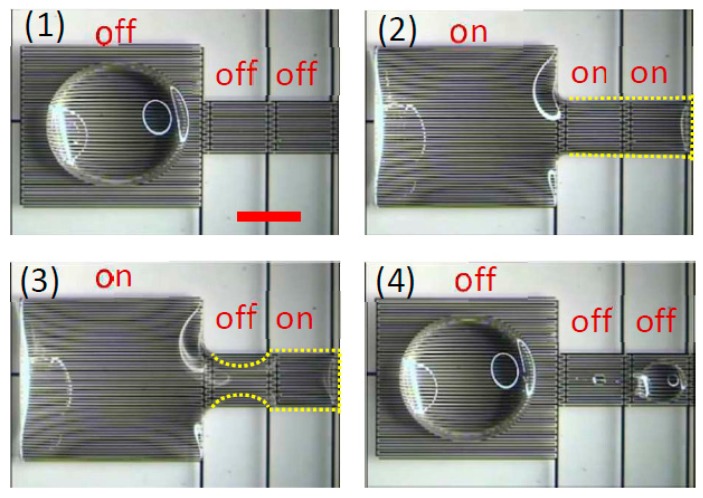
Droplet dispensing using dielectrowetting. (**1**) A 22-μL sessile droplet of propylene carbonate on the electrode pads. (**2**) The electrode pads are turned on, spreading the droplet. (**3**) The middle pad is turned off to pinch off the droplet. (**4**) All the pads are turned off and droplet separation is complete. Reproduced with permission from the original in study [93].

One of the most popular techniques in DMF is electrowetting-on-dielectric (EWOD). A droplet is placed between two plates, one of which contains a dielectric layer. A voltage difference across the droplet generates asymmetric droplet contact angles, thus creating a driving force. Switching the voltage difference in a timely manner moves the droplet [92,93,94]. Optoelectrowetting is a modified version of the EWOD technique, where the voltage switching is accomplished optically [95,96]. Recently, Geng et al. [93] reported a pioneering work regarding the use of the dielectrowetting [97] instead of EWOD to manipulate both conductive or non-conductive droplets. This concept removes the need for a top plate and provides easy access to the droplets. The principle operation of such a technique is shown in Figure 9.

#### 3.1.2. Dielectrophoretic Technique

**Figure 10:**
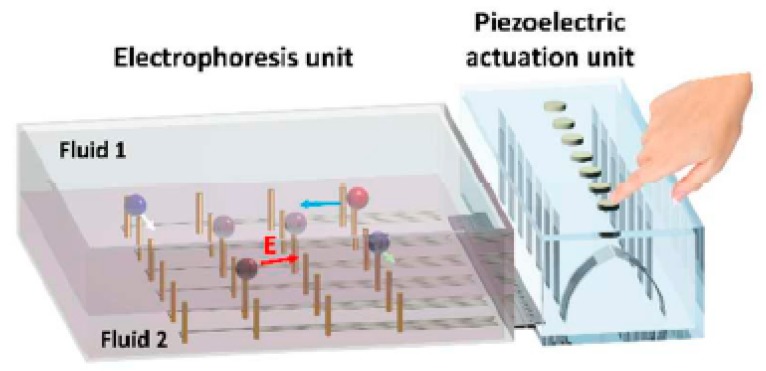
Schematic showing the finger-powered electrophoresis unit. The electrodes are connected to the piezoelectric actuation unit, which can be actuated by pressing on it. The electrophoresis unit contains both the electrode array and the droplets, suspended in a binary fluid. Reproduced with permission from the original in study [100].

The dielectrophoresis technique similarly uses electrostatic force, but the droplet itself acts as a dielectric [98,99]. In one of the most recent works, Iwai et al. [100] combine “finger-powered” microfluidics with piezoelectric elements to achieve dielectrophoretic droplet manipulation. The device harnesses the user’s mechanical input and converts it into electrostatic energy, which is then used to move the droplets suspended in fluids, Figure 10.

#### 3.1.3. Magnetic-Based Techniques

Instead of an electric field, a magnetic field can be applied to move droplets containing magnetite via magnetowetting [101]. The magnetic field generates a body force throughout the entire droplet. Displacing a permanent magnet under a ferrofluid droplet creates asymmetric contact angles and moves the droplet.

#### 3.1.4. Other Techniques

Droplets can be manipulated using other means such as surface acoustic waves (SAW) [102,103,104] or thermocapillary forces [105,106]. Acoustic energy is generated using a piezoelectric element and transferred to the droplet. As the SAW hit the droplet, energy is transferred onto the droplet, which causes it to de-pin from the surface and move. More energetic SAW can even cause droplets to nebulise. Unlike EWOD, most SAW devices need only one plate. On the other hand, a thermocapillary-based DMF device moves a droplet using capillary forces generated by surface tension gradients which arise from temperature differentials. Nguyen and Huang [107] evaluated the manipulation of droplets in long capillaries under a variable temperature field. In particular, they evaluated the initial behaviour of liquid motion under a transient temperature gradient, both analytically and experimentally.

### 3.2. Liquid-Marble-Based DMF

**Figure 11:**
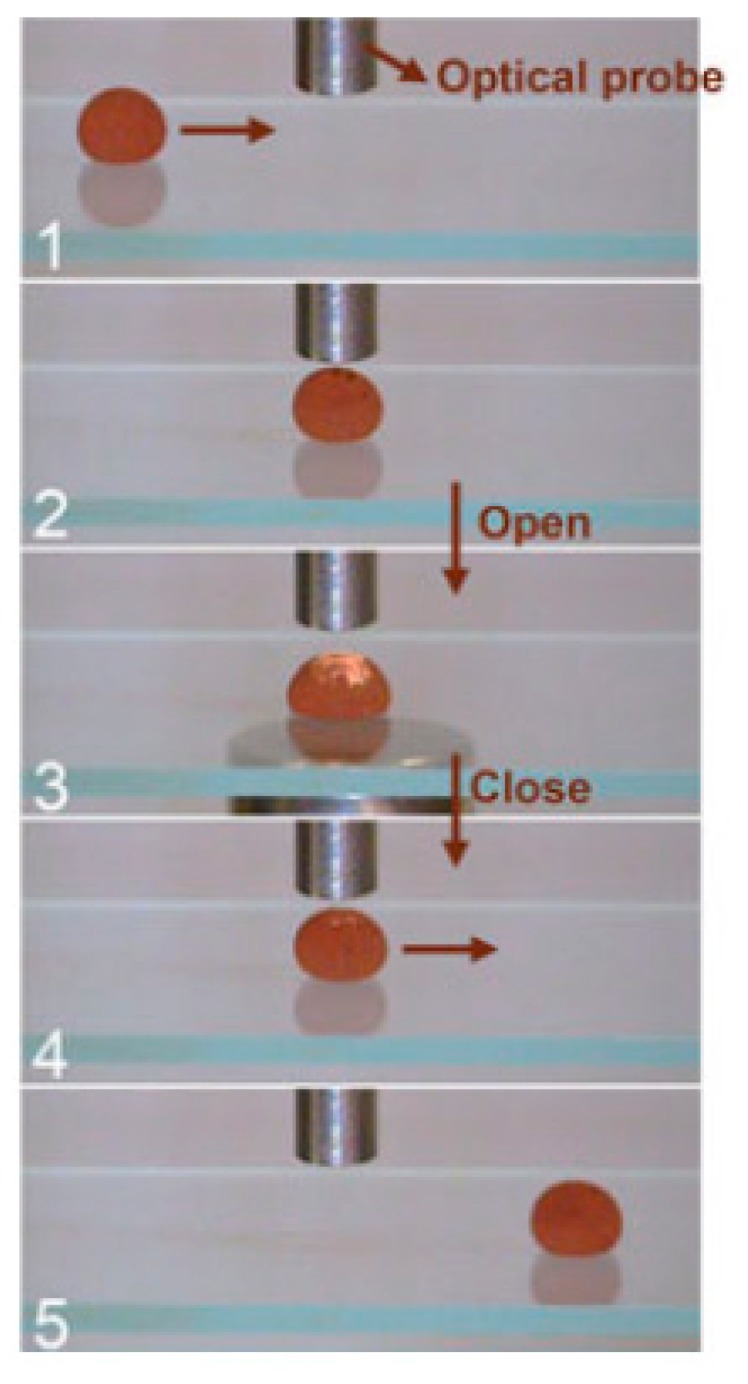
A magnetite-covered LM used as a miniature bioreactor. (**1**,**2**) The LM containing the reactants is moved towards the optical probe using a permanent magnet. (**3**) The coating of the LM can be “opened” to reveal its contents by increasing the magnetic field. (**4**,**5**) The coating opening process is reversed and the LM is moved away from the probe. Reproduced with permission (CC BY license) from original in study [120].

Another growing field in DMF is the use of liquid marble (LM) as the discrete platform. The LM is a small droplet encapsulated by a hydrophobic coating, which consists of a porous particle layer [108,109,110,111]. The hydrophobic and porous shell removes the need for surface treatment, as the droplet is physically isolated from its surroundings. An added benefit is that a LM is able to float on a liquid surface [112,113] and seemingly skid around with low friction [114,115]. As discussed by Ooi and Nguyen [116] in a comprehensive review paper, numerous techniques to manipulate the LM have been derived. Among the most popular techniques is manipulating a LM containing magnetite using a permanent magnet [117,118,119,120,121]. Zhao et al. [120] used an encapsulated LM driven by a permanent magnet as a bioreactor, as illustrated in Figure 11. Furthermore, a LM can be driven by thermo- [122,123] or soluto-capillary forces, and even carry its own propellant whilst doing so [124,125,126].

### 3.3. Advanced Digital Microfluidic Platforms

Recent advances in manipulating microdroplets predominantly involve EWOD-based devices. Researchers have pioneered the use of DMF in the immunoprecipitation process [127]. This concept was accomplished using an existing DMF device which combines both EWOD and magnetic manipulation of the droplet [128,129]. DMF has also been used for the first time in solid-phase micro extraction [130], as well as in high field nuclear magnetic resonance spectroscopy [131]. Nanostructure initiator mass spectrometry (NIMS) arrays can be integrated into an EWOD device to conduct enzyme screening, which potentially increases the throughput of the process [132]. On the cost-reduction front, a specially designed EWOD system has been manipulated using a smart phone to conduct chemiluminescence sensing [133].

However, liquid marble has recently shown its potential as an emerging digital microfluidic platform, especially for biological applications. The most prominent application of liquid marble has been cell culture and the ability to form three-dimensional spheroids due to its respirable and non-adhesive coating [134,135,136,137]. Liquid marble can be dehydrated to form hollow shells [138], which then is used for drug encapsulation and release [139]. Liquid marble can also be used as a microbioreactor, as it can accommodate liquid volumes across several orders of magnitude and still can be easily handled [118,140]. Recently, a spinning liquid marble has been used to improve mixing [141].

## 4. Conclusions and Perspectives

This paper summarises the most recent and advantageous advances in liquid handling modalities, using both continuous-flow and digital microfluidics. Due to the importance of mixing and separation in biological and chemical procedures, we confined the scope of continuous-flow microfluidics to these two topics. Mixing is an essential step in most lab-on-a-chip platforms, as sample preparation is required for a variety of biological and chemical assays. Diffusion-based mixing techniques fail to satisfy the recent demand for rapid and homogeneous mixing. Advances in two major mixing enhancement strategies, i.e., mixing with external energy sources, as well as complex channels geometry, were reviewed. Continuous-flow microfluidic separation also has the benefit of real-time monitoring and the potential for the integration with other continuous-flow processes. Based on the unique signature of the sample components, a suitable external force can be chosen for the separation process. Cutting-edge advances in continuous-flow microfluidic separation techniques, including magnetofluidics, inertial microfluidics, acoustofludics, dielectrophoretics and optofludics, were reviewed and discussed. Emerging applications of combined continuous-flow separation and mixing technologies for more advanced microfluidic platforms, such as diagnostic and therapeutic microbioreactors, lab-on-a-disc and microfluidic chromatography for protein purification, were introduced.

The second part of this paper was dedicated to digital microfluidics for handling microdroplets and liquid marbles. Droplet-based DMF techniques, such as electrowetting-on-dielectric (EWOD), dielectrophoresis, and magnetic methods were discussed. The applications of more advanced combinatorial DMF devices were also introduced. In addition, manipulation techniques for liquid marble as a microbioreactor were presented.

Recent advances in microfluidics indicate that more complex microfluidic structures, especially for mixing applications, could be fabricated with 3D printing. The design freedom provided by 3D printing will allow for novel designs, which to date cannot be obtained with planar micromachining techniques, such as soft lithography with PDMS. Microfluidic cell culture can be considered as the next-generation technique for biomedical and pharmaceutical applications. Liquid marble has emerged as a promising digital microfluidics platform. Continuous-flow microfluidics will continue to be used for applications that require high throughput. However, the problem of bulky external liquid delivery and the need of optical microscopy for characterisation makes continuous-flow microfluidics less suitable for applications with limited sample size. Digital microfluidics with droplets and liquid marbles is the solution for the problem of bulky external systems, as well as the relatively large sample volume. In the near future, we could expect more reports on this unique research area. As most recent works are only on the proof-of-concept of liquid-marble-based digital microfluidics, automated systems for creating liquid marble and the controlled manipulation of liquid marble, such as coalescence and splitting, are areas of interest for bringing this platform closer to practical use.
